# Printing Three‐Dimensional Refractory Metal Patterns in Ambient Air: Toward High Temperature Sensors

**DOI:** 10.1002/advs.202302479

**Published:** 2023-08-06

**Authors:** Jichuan Yu, Chuxiong Hu, Ze Wang, Yuankong Wei, Zhijin Liu, Qingang Li, Lei Zhang, Qiulin Tan, Xining Zang

**Affiliations:** ^1^ Department of Mechanical Engineering Tsinghua University Beijing 100084 China; ^2^ Beijing Key Laboratory of Precision/Ultra‐Precision Manufacture Equipments and Control Tsinghua University Beijing 100084 China; ^3^ Key Laboratory for Advanced Materials Processing Technology Ministry of Education Tsinghua University Beijing 100084 China; ^4^ State Key Laboratory of Dynamic Measurement Technology North University of China Tai Yuan 030051 China

**Keywords:** 3D printing, high temperature, laser sintering, refractory metal, wireless sensing

## Abstract

Refractory metals offer exceptional benefits for high temperature electronics including high‐temperature resistance, corrosion resistance and excellent mechanical strength, while their high melting temperature and poor processibility poses challenges to manufacturing. Here this work reports a direct ink writing and tar‐mediated laser sintering (DIW‐TMLS) technique to fabricate three‐dimensional (3D) refractory metal devices for high temperature applications. Metallic inks with high viscosity and enhanced light absorbance are designed by utilizing coal tar as binder. The printed patterns are sintered into oxidation‐free porous metallic structures using a low‐power (<10 W) laser in ambient environment, and 3D freestanding architectures can be rapidly fabricated by one step. Several applications are presented, including a fractal pattern‐based strain gauge, an electrically small antenna (ESA) patterned on a hemisphere, and a wireless temperature sensor that can work up to 350 °C and withstand burning flames. The DIW‐TMLS technique paves a viable route for rapid patterning of various metal materials with wide applicability, high flexibility, and 3D conformability, expanding the possibilities of harsh environment sensors.

## Graphical abstract

A direct ink writing and tar‐mediated laser sintering technique is presented to fabricate refractory metal‐based devices. The light absorption enhancement and anti‐oxidation function of tar allow metallic patterns to be sintered in ambient air with a low‐power laser. A wireless sensor that can endure high‐temperature and flaming conditions is demonstrated, highlighting the potential for sensing applications in harsh environments.

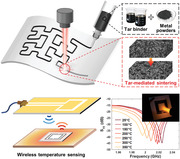

## 1 Introduction

Real‐time monitoring of critical environmental parameters is of great importance in extreme industrial scenarios including thermal power generation, oilwell drilling, and aerospace for improving reliability and preventing failures.^[^ ^1^ ^]^ The harsh environments, especially high temperature conditions, impose formidable challenges to the durability and longevity of the sensors.^[^ ^2^ ^]^ Refractory metals (such as molybdenum and tungsten) and their alloys are promising candidates for high‐temperature electronics due to their high‐temperature and corrosion resistance as well as excellent durability to thermal and mechanical stress.^[^ ^3^ ^]^ However, fabrication of refractory metal‐based devices is challenging and costly due to their high melting temperature and poor processability.^[^ ^4^ ^]^

State‐of‐the‐art additive manufacturing (AM) techniques offer new possibilities for engineering refractory metals,^[^ ^5^ ^]^ among which, powder bed‐based approaches including laser powder bed fusion and electron beam powder bed fusion are most widely studied.^[^ ^6^, ^7^ ^]^ Despite the advantages of high accuracy and the ability to produce complex shapes, powder bed‐based AM methods are not suitable for fabricating electronic devices due to the limitations of rigorous requirement for protective environments, dependence on high‐quality powder beds, and poor compatibility with other circuit manufacturing processes.^[^ ^8^ ^]^ Alternatively, ink extrusion‐based three‐dimensional (3D) printing, in which patterns are firstly printed by binder‐containing metallic ink followed by thermal treatments to reduce the additives and sinter the metallic structures, is an appealing option to create conductive metallic patterns with low cost and high flexibility.^[^ ^9^, ^10^ ^]^ While traditional furnace sintering methods involve long heating time with high energy cost,^[^ ^11^ ^]^ laser sintering, characterized by high resolution and high power density,^[^ ^12^ ^]^ has emerged as an effective approach to provide in situ thermal treatment for printed patterns, which has been employed to fabricate highly conductive circuits in copper, silver, and zinc.^[^ ^13^, ^14^, ^15^ ^]^ By controlling the laser parameters, tailored electrical performances can be obtained.^[^ ^16^ ^]^ However, metallic materials typically exhibit high reflectance to light ranging from visible to near infrared wavelengths, resulting in low energy utilization rate and limited heating temperature during the laser sintering process.^[^ ^17^ ^]^ This presents a significant challenge in meeting the sintering requirements for refractory metals. Moreover, increased laser radiation may lead to severe oxidation and overablation rather than better sintering results.^[^ ^18^ ^]^ Consequently, an efficient and economical fabrication method for refractory metal patterns remains to be unlocked.

**Figure 1:**
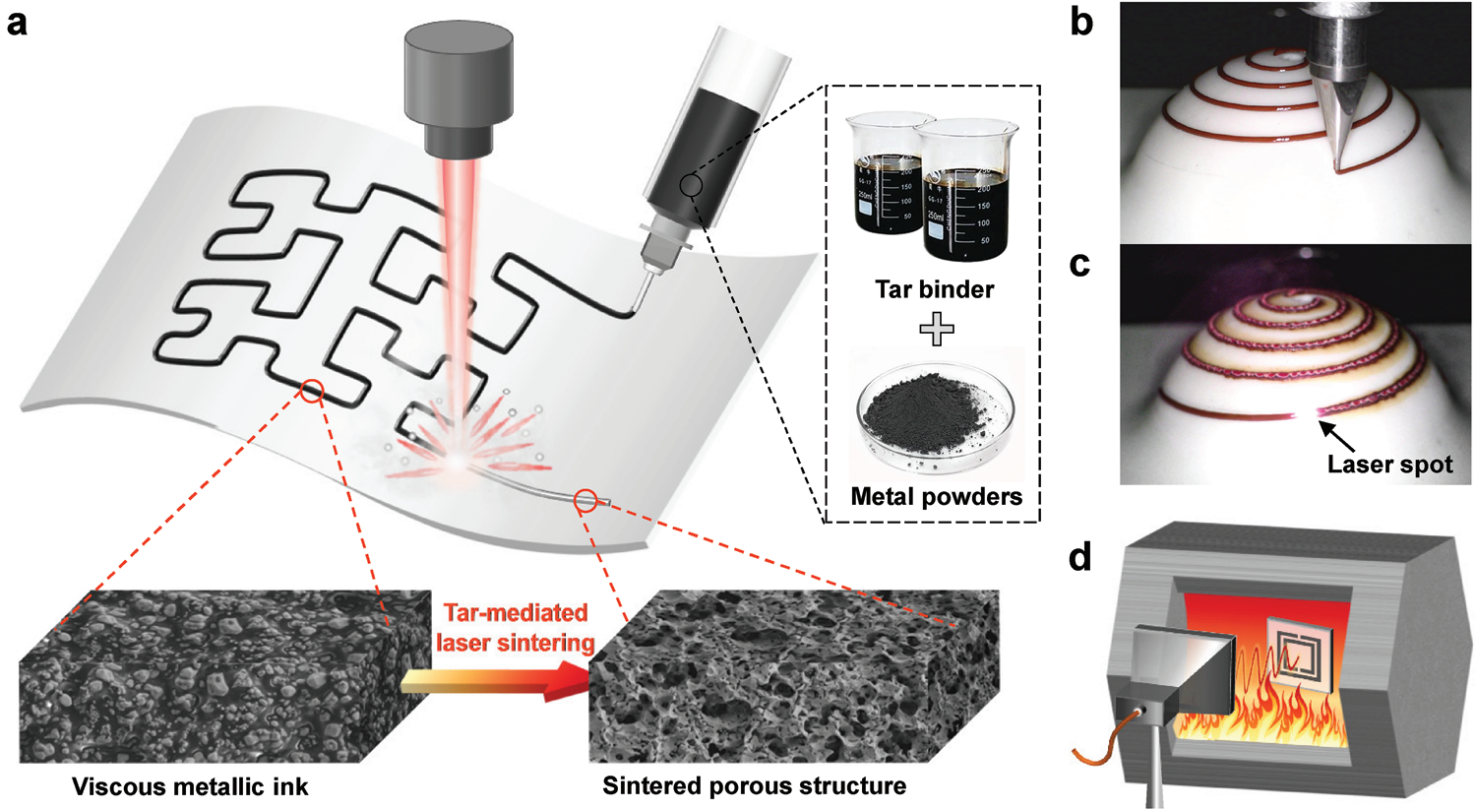
a) Schematic illustration of direct ink writing and tar‐mediated laser sintering (DIW‐TMLS). Photographs showing the b) printing and c) laser sintering processes of a conformal spiral pattern on a ceramic hemisphere. d) Schematic of a wireless sensor based on refractory metal pattern operating in high‐temperature environments.

In this paper, we present a direct ink writing and tar‐mediated laser sintering (DIW‐TMLS) technique to fabricate 3D metallic patterns in ambient atmosphere (**Figure** **1**a–c). Coal tar, a byproduct of coal distillation, contains rich chemical compounds mainly comprising aromatic hydrocarbons.^[^ ^19^ ^]^ Despite the abundant production, coal tar is commonly regarded as a hazardous waste that is underutilized and undervalued.^[^ ^20^ ^]^ We show that tar can serve not only as binder to prepare metallic inks with preferable rheological features and enhanced light absorption properties, but also as an effective antioxidant. The mediation of tar during laser sintering leads to extremely high local temperature inside the printed line, facilitating the formation of homogeneous porous metallic structure. Utilizing molybdenum (Mo, a typical refractory metal with high melting temperature of 2610 °C) and copper (Cu, a typical high light reflectivity metal) as representatives, we demonstrate that the printed patterns can be successfully sintered in ambient air via a low‐power infrared laser without oxidation. The morphology, composition, and electrical conductivity can be controlled by laser parameters within a wide processing window. By integrating the printing and laser sintering processes into a single motion platform (Figure S1, Supporting Information), self‐supported 3D metallic structures can be created. Applications including a fractal pattern‐based strain gauge and a conformal hemispherical antenna are presented to verify the flexibility and wide applicability of the DIW‐TMLS technique. Furthermore, we demonstrate a wireless temperature sensor based on Mo patterns that can stably work up to 350 °C and withstand burning flames, highlighting the potential of the proposed method to fabricate sensors for high temperature applications (Figure 1d).

## 2 Results and Discussion

### 2.1 Ink Preparation and Characterization

The metal‐tar ink is primarily a homogeneous mixture composed of coal tar, PVP (polyvinylpyrrolidone)‐NMP (*N*‐methyl‐2‐pyrrolidone) solution, along with micron‐sized metal powders. Coal tar mainly consists of polycyclic aromatic hydrocarbons (PAHs) and alkanes, the molecular weights of which lie in the range of 200 to 400 Da, as indicated by the mass spectrometry results (Figure S2 and Table S1, Supporting Information). The low H:C atom ratio suggests a high degree of unsaturation. The highly condensed organic components in tar result in a high viscosity, making it an ideal binder. In addition, the low attenuation coefficient and high light absorptivity of tar enable deeper penetration of the laser beam.^[^ ^21^ ^]^ PVP‐NMP solution facilitates a more uniform and stable dispersion of the metal powders,^[^ ^22^ ^]^ while also playing a role in anti‐oxidation by generating carboxylic acids as reducing agents under laser irradiation.^[^ ^23^ ^]^ Molybdenum (Mo) and copper (Cu) are selected as representatives for preparation of the inks. The Mo powders, which are produced by mechanical grinding techniques, show a wide particle size distribution and irregular powder morphology (Figure S3a, Supporting Information). In comparison, the adopted Cu powders, including both fine powders (1 µm) coarse powders (5 µm), have a narrower particle size distribution and better sphericity (Figure S3b, Supporting Information), which are produced by chemical reduction methods.

The compositions of the metal‐tar inks have been optimized to achieve a preferable rheological behavior. The viscosity, pre‐yield modulus as well as the yield stress of the ink show an upward trend with increasing metal content (Figure S4b,c, Supporting Information), enhancing its ability to retain the extruded shape. Ink prepared with fine powders has higher rheological indexes than ink prepared with coarse powders (Figure S4d,e, Supporting Information), which can be explained by the fact that the amount of particles and contacting neighbors per unit volume increases with the decrease of particle size, leading to more interparticle friction.^[^ ^24^ ^]^ In addition, the modulus and the yield stress of the ink can be further enhanced by utilizing multi‐sized powders (Figure S4f, Supporting Information), as higher packing density can be achieved.^[^ ^25^ ^]^ As a result of optimization, the Cu‐tar ink contains 85.7 wt% of metal using a mixture of 1 µm fine powders and 5 µm coarser powders with mass ratio 1:1, while the Mo‐tar ink contains 77.3 wt% of metal utilizing powders with a particle diameter less than 5 µm.

**Figure 2:**
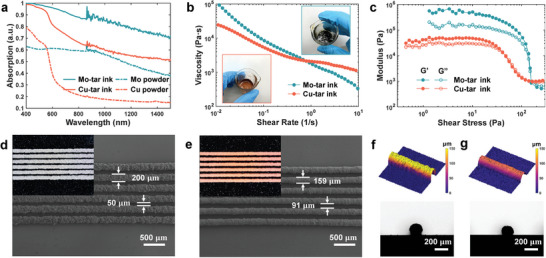
Characterization of the metal‐tar inks. a) Comparison of absorption spectrum between metal‐tar inks and pure metal powders. b) Apparent viscosity as a function of shear rate showing the shear‐thinning behavior. c) Storage modulus (*G*’) and loss modulus (*G*’’) as functions of shear stress showing the yield behavior. Scanning electron microscopy (SEM) and optical images of the printed d) Mo‐tar and e) Cu‐tar filaments shows high printing resolution. Three‐dimensional (3D) surface profiles and cross‐section views of the printed f) Mo‐tar and g) Cu‐tar filaments.

The well‐mixed metal‐tar inks show a significant increase in light absorbance from visible to near‐infrared range compared to pure powders (**Figure** **2**a), which augments the efficiency of photon‐to‐thermal energy conversion. This distinctive feature empowers the metallic ink to be heated to a higher temperature when subjected to laser irradiation. The prepared inks exhibit a shear thinning behavior (i.e., the apparent viscosity decreases with increasing shear rate) as shown in Figure 2b, and show the properties of yield‐stress fluids as can be seen in Figure 2c. Due to wider particle size distribution and irregular powder morphology, Mo powders tend to hinder the flow of ink under low shear stress, resulting in higher viscosity at low shear, higher pre‐yield modulus and yield stress. Utilizing a pneumatic dispenser (Figure S1, Supporting Information), the prepared Mo‐tar ink can be printed smoothly with a nozzle of 200 µm inner diameter (Figure 2d), while Cu‐tar ink can be printed with a 150 µm line diameter (Figure 2e). The printed filaments can well maintain the extruded shape without slumping, as shown in Figure 2f,g. The cross‐section of printed Cu‐tar filament shows a semicircular shape, while the Mo‐tar filament exhibits a higher height to width ratio, which can also be attributed to the differences in the size distribution and morphology of powders.

### 2.2 Laser Sintering of Metal‐Tar Inks

A 976 nm continuous‐wave infrared laser with a focusing spot of 105 µm diameter is utilized for in‐situ sintering of printed patterns. The enhanced light absorption property of the ink enables us to choose a low laser power (≤10 W). When subjected to laser irradiation, the alkanes and PAHs in tar are transformed into amorphous carbon.^[^ ^20^, ^26^ ^]^ The above reactions result in the formation of a reducing atmosphere on the surface of the ink. Consequently, a slower laser scanning rate (≤100 mm min^−1^) can be adopted, which is able to generate a higher internal temperature in the metallic ink to achieve sintering without oxidation. The metal filaments are printed using a nozzle with an inner diameter of 250 µm for convenience of the subsequent characterization. When lasing at focus, the irradiated areas experience excessive ablation, resulting in the formation of craters on the surface and severe balling of melted droplets. However, the surrounding areas are not sufficiently sintered (Figure S5a,c, Supporting Information). On the contrary, defocusing the laser so that the spot fully covers the filament produces a more uniform energy distribution and thereby more homogenous and complete sintering results (Figure S5b,d, Supporting Information).

**Figure 3:**
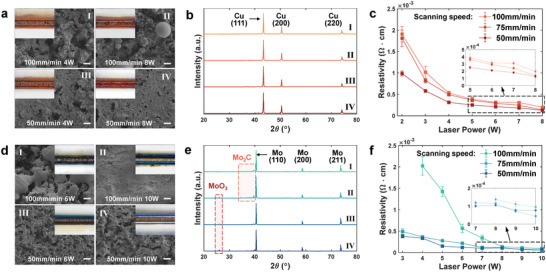
Effects of laser scanning parameters on microstructures, phases, and electrical conductivity of the sintered pattern. a) Scanning electron microscopy (SEM) and optical images of the sintered Cu‐tar filaments under different laser powers and scanning rates. Scale bar: 10 µm. b) X‐ray diffraction (XRD) patterns of the sintered Cu‐tar filaments under different laser parameters. Each curve corresponds to a subgraph in (a). c) Variation of resistivity of the sintered Cu‐tar pattern with laser power and scanning rate. d–f) SEM and optical images (Scale bar: 20 µm), X‐ray diffraction (XRD) patterns, and resistivity of sintered Mo‐tar filaments under different laser parameters.

Optical imaging, scanning electron microscopy (SEM) and X‐ray diffraction (XRD) are utilized to characterize the sintering results. For Cu‐tar ink, scanning at a speed of 100 mm min^−1^ induces a rough surface morphology. A laser power as low as 4 W is able to achieve partial sintering (**Figure** **3**a). When the laser power is increased to 8 W, metallic droplets occur on the surface. Such balling phenomenon is also frequently seen in powder bed fusion processes, which is usually attributed to melt splashes.^[^ ^27^ ^]^ Due to the inadequate consumption of tar and low solid carbon solubility in Cu, amorphous carbon precipitation is observed on the surface. Simply increasing the laser power cannot refine the surface morphology. Instead, it leads to more severe over‐ablation. By employing a lower scanning speed, a more complete and uniform sintering can be achieved, resulting in the formation of a flattened and densified structure. A scanning speed within the range of 50 to 75 mm min^−1^ is ideal for optimal sintering (Figure S6, Supporting Information). In addition, no significant oxides are observed at different laser parameters as indicated by the XRD patterns in Figure 3b, validating the anti‐oxidation function of tar. The resistivity of the sintered Cu pattern decreases as the laser power increases and the scanning rate decreases, reaching a minimum of 1.27 × 10^−4^ Ω cm at the laser power of 8 W and scanning rate of 50 mm min^−1^, as can be seen in Figure 3c.

Mo‐tar ink, which exhibits near‐blackbody absorbance characteristics (Figure 2a), can also be successfully sintered with a laser power no more than 10 W, despite the high melting temperature of molybdenum. Intermittent melted features can be observed under the scanning parameter of 100 mm min^−1^ and 6 W, while a fully melted layer is formed when the power is increased to 10 W as shown in Figure 3d. Figure S7 (Supporting Information) provides a more comprehensive presentation of the evolution in the surface microstructures. Tar in the ink is not fully consumed at a scanning speed of 100 mm min^−1^. The oversaturation of carbon leads to the formation of Mo_2_C phase (Figure 3e). It can be inferred from the Mo‐C phase diagram^[^ ^28^ ^]^ that the internal temperature of the ink reaches at least 1600 °C. The presence of Mo_2_C phase does not significantly deteriorate the electrical performance of the pattern.^[^ ^29^ ^]^ When the scanning speed descends to 50 mm min^−1^, a porous surface morphology is obtained, with the organic components agents fully removed. No evident Mo_2_C phase appears in the XRD pattern, instead, a small amount of oxide occurs due to the depletion of tar. The resistivity shows a tendency to decrease with decreasing scanning rate and increasing power (Figure 3f). However, there is a rebound at the scanning speed of 50 mm min^−1^ as the power increases, which may be attributed to the slight oxidation as verified by the XRD pattern. A minimum resistivity of 4.47 × 10^−5^ Ω cm is achieved at the scanning parameter of 75 mm min^−1^ and 10 W, which is only 8 times of bulk Mo (5.34 × 10^−6^ Ω cm). It is worth noting that our work enables the sintering of irregular micron powders into homogenous and highly conductive metallic structures, which represents a significant higher feasibility compared to previously reported studies that require high‐quality nanosized powders.^[^ ^13^, ^14^, ^15^, ^23^ ^]^

### 2.3 Sensors Characterization

**Figure 4:**
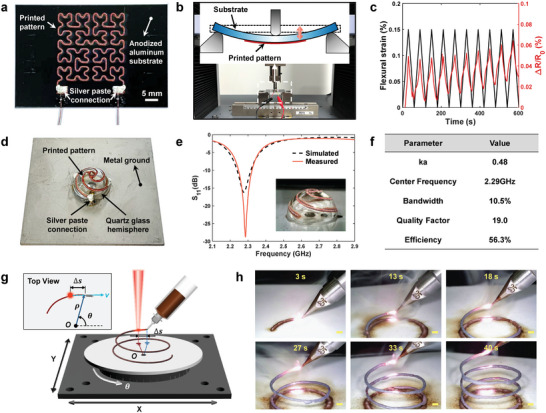
Metallic patterns fabricated by direct ink writing and tar‐mediated laser sintering (DIW‐TMLS) using Cu‐tar ink for various applications. a) Photograph of a fractal pattern‐based strain gauge printed on an anodized aluminum substrate. b) Schematic of three‐point bending test for the strain gauge. c) Resistance change of the strain gauge in response to the cyclic flexural strain. d) Photograph of a conformal spiral antenna printed on a quartz glass hemispherical substrate. e) Simulated and measured reflection coefficient (S_11_) of the hemispherical antenna. f) Key performance indexes of the hemispherical antenna. g) Schematic of creating self‐supported 3D structures by mid‐air sintering. h) Photographs showing the printing process of a freestanding helical structure. Scale bar: 1 mm.

DIW‐TMLS enables rapid production of metallic patterns with high resolution on various substrates for a wide range of applications. **Figure** **4**a presents a fractal pattern‐based strain gauge. The pattern is printed with Cu‐tar ink on an anodized aluminum substrate and has a minimum radius of curvature of only 0.875 mm at the inflections. The porous nature of the sintered structure makes its resistance sensitive to mechanical stress, while the fractal design enhances the stretchability of rigid circuits.^[^ ^30^ ^]^ Three‐point bending tests are conducted on the fabricated strain gauge (Figure 4b). The resistance of the pattern shows a trend consistent with the flexural strain under the bending‐releasing cycles (Figure 4c).

Next, in order to verify the feasibility to print on 3D curvilinear surfaces, a conformal hemispherical antenna is demonstrated, as shown in Figure 4d. A multi‐arm spiral pattern is printed on a quartz glass hemisphere with a radius of 10 mm. One of the arms is electrically connected to a SMA connector, while the other arms are attached to the metal ground. By tuning the numbers of arms and turns of each arm, the antenna is matched to have a 50 Ω impedance at the operating frequency. Detailed design parameters are provided in Figure S8 (Supporting Information). Compared with traditional planar designs, volumetric antennas offer miniaturization benefits and better electromagnetic performance.^[^ ^30^ ^]^ As can be seen in Figure 4e,f, the fabricated antenna has a center frequency of 2.29 GHz with the electrical size *ka* < 0.5 (where *k = 2π/λ* is the wavenumber and *a* is the minimum radius of a sphere that circumscribes the antenna), which can be considered as an electrically small antenna (ESA). The high bandwidth (BW, 10.5%) and low quality factor (19.0) is preferable for ESAs, indicating a high ratio of energy radiated to energy stored. A radiation efficiency of 56.3% is reasonable due to the small electrical size and relatively higher resistivity compared to pure copper. In addition, the performance characteristics of hemispherical antennas fabricated by different 3D patterning techniques (including direct transfer patterning,^[^ ^31^ ^]^ pad printing,^[^ ^32^ ^]^ and silver ink writing^[^ ^33^ ^]^) are listed in Table S2 (Supporting Information). Unlike the existing methods that involve multiple processing steps and lengthy curing time, DIW‐TMLS integrates patterning and sintering into a single platform, with total processing time less than 5 min (Video S1, Supporting Information).

Moreover, we show that laser sintering can be implemented simultaneously with printing to create self‐supported 3D structures. To achieve this, the syringe is tilted to an angle of approximately 45°, and the laser spot is aligned with the nozzle, as illustrated in Figure 4g. The printed ink is immediately sintered by the laser spot following closely behind. By precisely controlling the rotation angle of the rotary stage, the laser‐nozzle line remains consistently parallel to the tangent of the trajectory,^[^ ^13^ ^]^ which enables the fabrication of curved patterns in 3D space. Figure 4h and Video S2 (Supporting Information) present the mid‐air sintering process of a freestanding helical structure which spans three turns in space. The sintered structure rests approximately 0.3 to 0.4 mm below the trajectory of the nozzle (Figure S9, Supporting Information), due to the existence of a gap ∆s between the laser spot and the nozzle. Ideally, reducing ∆s can improve the printing accuracy. However, a heat dissipation region emerges along the printed line when subjected to laser heating, in which the rheological behaviors are changed due to the increase in temperature. To ensure minimal impact on ink extrusion, ∆s is set to 1 mm in the experiment. By incorporating DIW‐TMLS into motion systems with more freedom (such as 5‐axis systems or robotic manipulators),^[^ ^34^, ^35^ ^]^ it is anticipated that more complex 3D structures can be created.

After mid‐air sintering, the line width of the printed filament expands to approximately twice its original size (Figure S10a,b, Supporting Information). There are several reasons for this. Firstly, the printing speed is set slightly slower than the ink extrusion speed to prevent breakage, which causes accumulation of ink at the laser spot and leads to volume increase. Secondly, compared to the ink adhered to the substrate, the suspending ink exhibits a much slower heat dissipation rate, leading to a more pronounced accumulation of heat. To avoid excessive ablation, a relatively low laser power of 4 W is selected. Consequently, mid‐air sintering process involves longer heating and cooling stages. During the heating stage, tar is fully consumed, while the ink on the back side of the laser irradiation undergoes sinking due to gravity. These combined factors contribute to the formation of a porous sintered structure (Figure S10c,d, Supporting Information).

**Figure 5:**
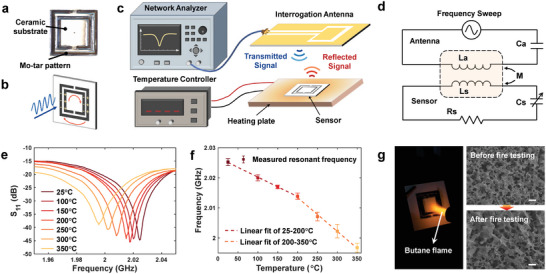
Molybdenum pattern‐based wireless high‐temperature sensor. a) Photograph of the temperature sensor with a split‐ring resonator (SRR) pattern printed on ceramic substrate. b) Schematic showing the charge distribution in the metal rings under excitation of polarized electromagnetic waves. c) Schematic of wireless temperature sensing. d) Equivalent circuit of the wireless sensing system. e) Measured reflection coefficient (S_11_) spectra at different temperatures. f) Variation of the resonant frequency of the sensor with respect to temperature. g) Fire resistance testing of the sensor. The scanning electron microscopy (SEM) images show no significant change in the surface morphology after accumulated 30 s of flame treatment. Scale bar: 20 µm.

To highlight the ability of DIW‐TMLS to produce electronics for harsh environment applications, we demonstrate a wirelessly inquired temperature sensor. Specifically, a split‐ring resonator (SRR) pattern (i.e., two interleaved metallic rings with two opposite gaps) is printed on a planar ceramic substrate using Mo‐tar ink (**Figure** **5**a). The entire structure functions as a LC oscillator that has an absorption peak of power at the resonant frequency when excited by a polarized electromagnetic wave (Figure 5b). The resonant frequency is negatively related to the dielectric constant of the substrate. As the environmental temperature increases, the dielectric constant of the substrate increases, leading to a decrease in the resonant frequency.^[^ ^36^ ^]^ Specific design parameters of the sensor as well as simulation results are provided in Figures S11 and S12 (Supporting Information). The wireless temperature sensing platform is depicted in Figure 5c, where the sensor is placed on a heating plate and wirelessly inquired by an interrogation antenna that is connected to a network analyzer. The interrogation antenna and the sensor are near‐field coupled, which can be represented by an inductively coupled circuit as shown in Figure 5d. The resonant frequency of the sensor can be extracted by one‐port scattering analysis of the network analyzer.^[^ ^37^ ^]^ The measured reflection coefficient spectra at different temperatures are shown in Figure 5e. The resonant frequency of the sensor is reduced from 2.025 to 1.996 GHz as the temperature rises from 25 °C to 350 °C. The measurement is repeated for four temperature cycles and the results show high reproducibility. The change of resonant frequency with respect to temperature is shown in Figure 5f, which can be approximately described by a segmented linear relationship. The sensitivity of the sensor is 61.84 kHz °C^−1^ in the temperature range of 25–200 °C and 112.5 kHz °C^−1^ in the temperature range of 200–350 °C, respectively. In order to further verify the tolerance to extreme environments, the sensor is heated by a butane spray gun for 10 s, which is repeated for three times. No significant change in the morphology of the sintered metallic structure is observed after flame heating (Figure 5g), highlighting the remarkable ability to withstand extreme environments.

## 3 Conclusion

In summary, we present a DIW‐TMLS technique for 3D rapid fabrication of metallic conductive patterns in ambient atmosphere. By introducing tar as binder, viscous metallic inks with enhanced light absorptance and anti‐oxidation property are developed. Both copper and molybdenum‐based ink can be sintered in ambient air without oxidation using an infrared laser with power less than 10 W, which is nearly 2 orders of magnitude lower than that required for selective laser sintering.^[^ ^6^ ^]^ The sintering mechanism is investigated with regard to the morphology, composition and electrical properties. With optimized laser parameters, the resistivity of sintered molybdenum pattern reaches the same order of magnitude as that of bulk metal. A fractal pattern‐based strain gauge as well as a conformal hemispherical antenna is demonstrated, validating the high flexibility and wide applicability of the DIW‐TMLS. Moreover, 3D freestanding architectures can be created by implementing printing and laser sintering simultaneously. Finally, a molybdenum pattern‐based wireless temperature sensor is developed, which is capable of operating under high temperature up to 350 °C and enduring flaming environments. The proposed DIW‐TMLS technique breaks through the processing limits of refractory metals, offering a viable path for rapid prototyping of high temperature sensors.

## 4 Experimental Section

### Materials

Coal tar was provided by Beijing Anshan Steel Research Institute. Copper powders (1 and 5 µm), PVP (K17, MW = 8000) powders and NMP were purchased from Jiangsu Aikon Biopharmaceutical R&D Co., Ltd. Molybdenum powders (<5 µm) were purchased from Beijing Zhongke Yannuo Technology Co., Ltd. Ethylene glycol butyl ether (EGBE) was purchased from MERYER Co., Ltd, China.

### Ink Preparation

For Cu‐tar ink, PVP powders were first dissolved into NMP with mass ratio 1:1 using a magnetic stirrer at 60 °C. Then 12 g 1 µm copper powders and 12 g 5 µm copper powders were dispersed into the mixture of 3 g coal tar and 1 g PVP‐NMP solution using an electric lab mixer. The ink was then mixed with a vacuum planetary centrifugal mixer (Shenzhen integrity Laser Co., Ltd.) at a speed of 1000 rpm for 30 min. Finally, the ink was transferred into a 5 mL syringe and centrifuged for 15 min in order to remove the air bubbles. For Mo‐tar ink, 16 g molybdenum powders were dispersed into the mixture of 3 g coal tar and 1.5 g PVP‐NMP (mass ratio 2:3) solution. Then 0.2 g EGBE was added into the ink as surfactant. The remaining steps were the same as Cu‐tar ink.

### Pattern Printing and Laser Sintering

The syringe was mounted on a 4‐axis (three translation axes plus one rotary axis) precision motion stage (Figure S1, Supporting Information), and connected to a pneumatic dispenser (DS‐982A, Taiwan Tech. & Material). The applied air pressure was set to 0.7 MPa. The ink was printed using stainless steel tapered nozzles with inner diameter ranging from 150 to 300 µm. Correspondingly, the printing speed ranged from 45 to 180 mm min^−1^. The substrate was mounted on a ceramic heating tablet, and was heated to about 80 °C after printing in order to remove the solvent. A 976 nm continuous wave diode laser (BWT Beijing Ltd.) with maximum power of 30 W was connected to the focusing lens by means of an optical fiber to obtain a minimum spot with a diameter of 105 µm. In the laser sintering process, the laser was defocused so that the spot is slightly larger than the printed linewidth in order to fully cover the printed line.

### Rheological Characterization

The rheological properties of the inks were measured using a rotational rheometer (MCR302, Anton Paar GmbH) equipped with a 25 mm‐diameter parallel plate fixture. Viscosities were measured via shear rate sweep test (0.01–10 s^−1^). Storage and loss modulus were measured via oscillatory stress sweep test (0.1–500 Pa) at a fixed frequency of 1 Hz. All experiments were conducted at room temperature.

### Material Characterization

The optical images were collected using a handheld microscope (Dino‐Lite). The microstructures were characterized using SEM (Zeiss Gemini 300) operated at 5 kV. XRD patterns were tested using Rigaku‐S2 X‐ray diffractometer. The resistance was measured by a precision source measure unit (SMU, Keysight B2912A) using the four‐probe method (Cascade EPS150FA probe station). The surface profile of the printed lines was measured using confocal laser scanning microscope (CLSM, Zeiss LSM 900). The chemical composition of tar was analyzed based on MALDI‐TOF mass spectrometry (AXIMA Performance, Shimadzu).

### Strain Gauge Fabrication and Measurement

A 4th order Hilbert's curve pattern^[^ ^30^ ^]^ (26 × 26 mm) was printed on an anodized aluminum substrate (40 × 60 mm) with a thickness of 0.8 mm. The pattern was lased at a power of 5 W and scanning rate of 70 mm min^−1^, and then electrically connected to the copper wires using silver conductive paste. The three‐point bending test was performed on a universal material testing machine (Shimadzu AGX‐V). The electrical signals were measured by a precision SMU (Keysight B2912A).

### Hemispherical Antenna Fabrication and Measurement

The hemispherical antenna was designed and simulated in the commercial software Ansys HFSS. The conformal spiral pattern was printed on a quartz hemisphere shell with 10 mm radius. A tilt angle of about 30° is selected for the nozzle during printing for better conformability. The pattern was then sintered at a laser power of 5 W and scanning rate of 70 mm min^−1^. One of the arms was electrically connected to the inner conductor of a coaxial transmission line using silver conductive paste, while the other arms were attached to the ground plane. The reflection coefficient was measured using a vector network analyzer (Agilent E5071C). The radiation efficiency was measured following the Wheeler cap method.^[^ ^38^ ^]^ The half‐power BW is defined as the ratio of the frequency range between the two half power points (reflection coefficient of −3 dB) to the center frequency, and the quality factor (*Q*) is inverse proportional to the BW, which is expressed as:^[^ ^34^ ^]^

(1) $$Q=\frac{5.828-1}{BW\cdot \sqrt{5.828}}$$

### Freestanding Structure Fabrication

A ceramic substrate was mounted on a stacked *X*–*Y*‐rotary stage (Figure S1, Supporting Information). The center of rotation was calibrated to coincide with the laser spot at the initial position. A tilt angle of 45° was selected for the nozzle during printing. The laser spot and the nozzle were aligned along the *X*‐axis with a gap of about 1 mm. Both the laser spot and nozzle move at the same speed of 120 mm min^−1^ with respect to the substrate, and a laser power of 4 W was adopted.

### Wireless Temperature Sensor Fabrication and Measurement

The SRR pattern was designed and simulated in the commercial software Ansys HFSS. The pattern was printed on a planar ceramic substrate with 0.5 mm thickness using Mo‐tar ink with nozzle of 300 µm inner diameter, and then lased at a power of 7 W and scanning rate of 70 mm min^−1^. A coplanar waveguide‐fed microstrip antenna with center frequency of 2.4 GHz (Figure S11, Supporting Information) was utilized to wirelessly interrogate the sensor. The reflection coefficient was measured using a vector network analyzer (Agilent E5071C).

## Conflict of Interest

The authors declare no conflict of interest.

## Supporting information

Supporting Information

Supplemental Video 1

Supplemental Video 1

## Data Availability

The data that support the findings of this study are available from the corresponding author upon reasonable request.
