# Supplementary material for: Printing Three‐Dimensional Refractory Metal Patterns in Ambient Air: Toward High Temperature Sensors
Source: Adv Sci (Weinh). 2023 Aug 6;10(31):2302479. doi: 10.1002/advs.202302479 (PMC10625119; doi:10.1002/advs.202302479)
Supplement: Supplementary file 1 — Supporting Information [file ADVS-10-2302479-s002.pdf]

ADVANCED SCIENCE Open Access

Supporting Information

for *Adv. Sci.*, DOI 10.1002/advs.202302479

Printing Three-Dimensional Refractory Metal Patterns in Ambient Air: Toward High Temperature Sensors

Jichuan Yu, Chuxiong Hu, Ze Wang*, Yuankong Wei, Zhijin Liu, Qingang Li, Lei Zhang, Qiulin Tan and Xining Zang**

Supporting Information

Printing Three-dimensional Refractory Metal Patterns in Ambient Air: Toward High Temperature Sensors

Jichuan Yu, Chuxiong Hu, Ze Wang*, Yuankong Wei, Zhijin Liu, Qingang Li, Lei Zhang, Qiulin Tan, Xining Zang**

DIW-TMLS Equipment

The direct ink writing and tar-mediated laser sintering processes are implemented on a 4-axis precision motion stage driven by stepper motors (Shinano Kenshi, Japan), as shown in **Figure S1(a)**. The three translational axes (X/Y/Z) have a stroke of 100 mm and resolution of 1 μm, while the rotary axis (θ) has a resolution of 0.005˚. The syringe filled with metal-tar ink is connected to a pneumatic dispenser (DS-982A, Taiwan Tech. & Material, **Figure S1(b)**), which provides a maximum pressure of 0.7MPa. The extrusion speed can be estimated by the Poiseuille's Law: [1]

$$\bar{V} = \frac{R^2 \Delta P}{8\tilde{\eta} L} \qquad (S1)$$

where $\tilde{\eta}$ is the extrapolated viscosity of the ink, ΔP is the pressure drop in the nozzle of length L and radius R. The inner diameter of the printing nozzle ranges from 150 μm to 300 μm, and the corresponding extrusion speed varies between 45 mm/min and 180 mm/min. The printing speed is set to match the estimated extrusion speed in order to obtain the desired printing linewidth. A 976 nm continuous wave diode laser (BWT Beijing Ltd., **Figure S1(c)**) with maximum power of 30 W is adopted to sinter the printed pattern. The laser beam is transported by an optical fiber and focused by the focusing lenses mounted next to the syringe. The printing nozzle and the laser spot are aligned by adjusting the manual microstage. The ceramic heating plate is placed under the substrate for preheating. The air nozzle is placed next to the motion stage and activated during the laser sintering process to blow away the spatter.

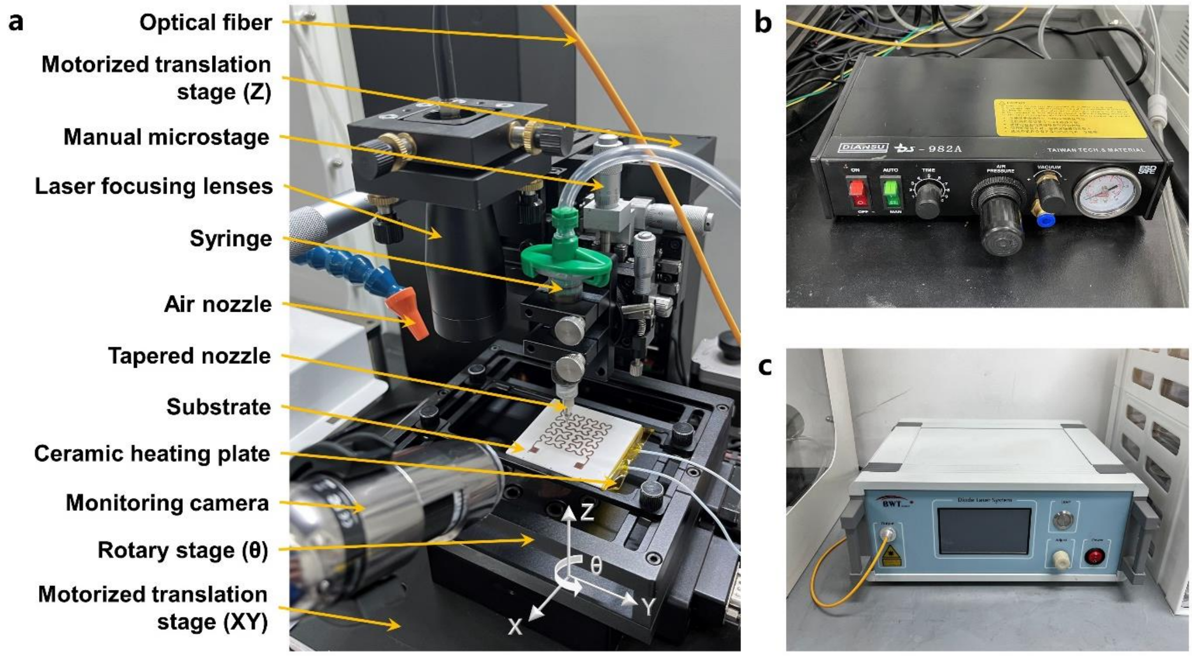

Figure S1. Photograph of the DIW-TMLS equipment. (a) Motion stage. (b) Pneumatic dispenser. (c) Diode laser system.

Composition analysis of tar

Matrix-assisted laser desorption ionization time-of-flight mass spectrometry (MALDI-TOF MS) is employed to identify the chemical composition of tar. As can be seen in **Figure S2**, the main signals are distributed mainly in the mass range between 200 and 400 Da. The mass spectrum is comprised of a series of peak clusters, where several subordinate peaks are distributed around their predominant peaks, indicating that the composition of tar is extremely rich. For simplicity, only the representative predominant peaks are examined. **Table S1** lists 8 polycyclic aromatic hydrocarbon (PAH) molecules that are matched to the predominant peaks. Peaks with odd molecular weights marked in **Figure S2** may contain nitrogen atoms and are not further considered. Since MALDI-TOF MS cannot provide specific structure information, only possible structures corresponding to the molecular formulas are provided. However, the results are credible because the differences in molecular weights between the listed PAH compounds are either 24 Da or 26 Da, satisfying the 24/26 rule (new aromatic cores are formed by incorporating C2 or C2H2 units, introducing a mass difference gap of 24/26 Da).[2] There are also a large amount of subordinate peaks that have certain mass increments to the listed ones, which can also be attributed to PAHs that have the same aromatic core but additional functional groups or side chains.[3] The abundance of PAHs results in a high degree of unsaturation, making tar an ideal antioxidative medium for laser sintering.

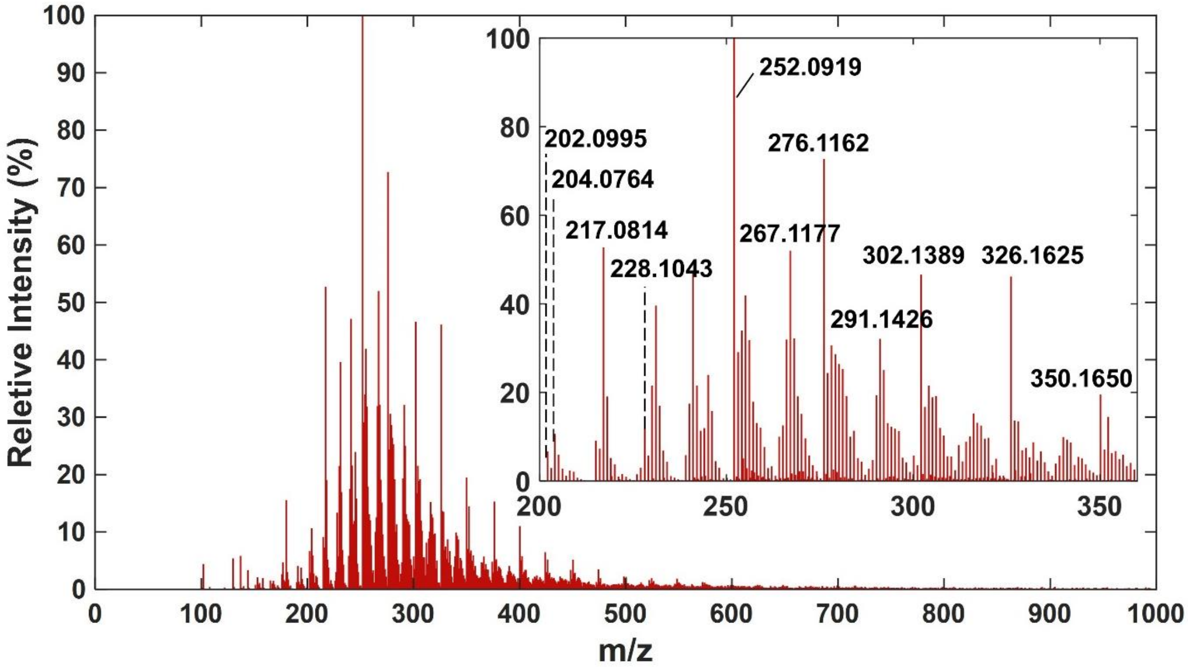

Figure S2. MALDI-TOF mass spectrum of tar (inset: magnified region between 200 and 360 Da).

Table S1. Molecular formulas and structures of PAH components identified from MALDI-TOF MS.

m/z	Formula	Potential PAH compound	m/z	Formula	Potential PAH compound
202	$C_{16}H_{10}$	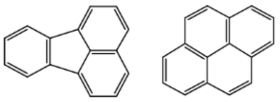	276	$C_{22}H_{12}$	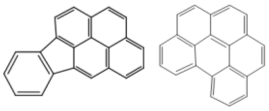 ...
204	$C_{16}H_{12}$	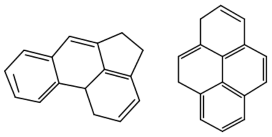	302	$C_{24}H_{14}$	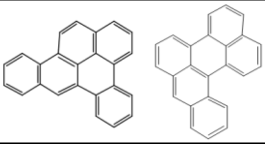 ...
228	$C_{18}H_{12}$	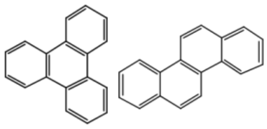 ...	326	$C_{26}H_{14}$	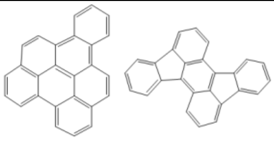 ...
252	$C_{20}H_{12}$	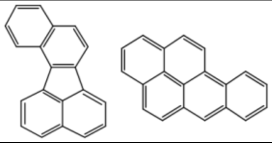 ...	350	$C_{28}H_{14}$	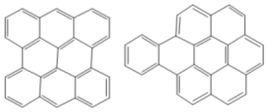 ...

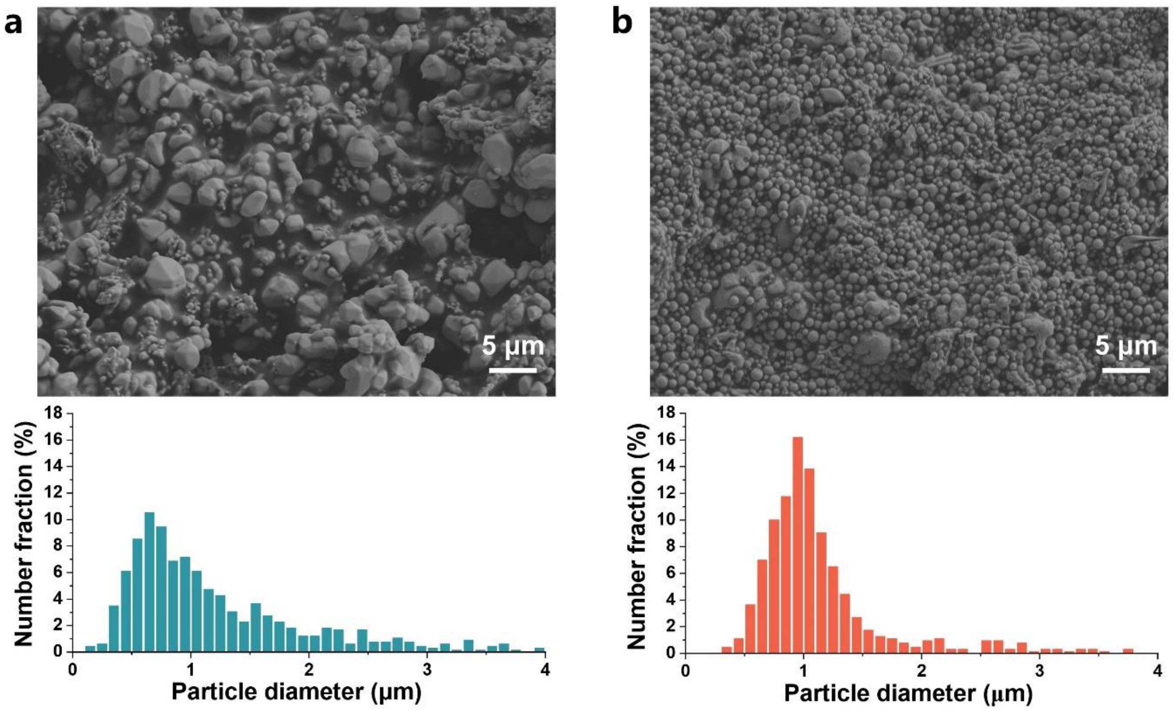

Figure S3. SEM images and particle size distributions of a) Cu-tar ink and b) Mo-tar ink.

a

Ink	Composition	Ink	Composition
**Mo-A**	tar (3 g) + Mo powders (13 g)	**Cu-A**	tar + coarse Cu powders (5 μm)
**Mo-B**	tar (3 g) + Mo powders (14.5 g)	**Cu-B**	tar + fine Cu powders (1 μm)
**Mo-C**	tar (3 g) + Mo powders (16 g)	**Cu-C**	tar + coarse Cu powders (5 μm) + fine Cu powders (1 μm)

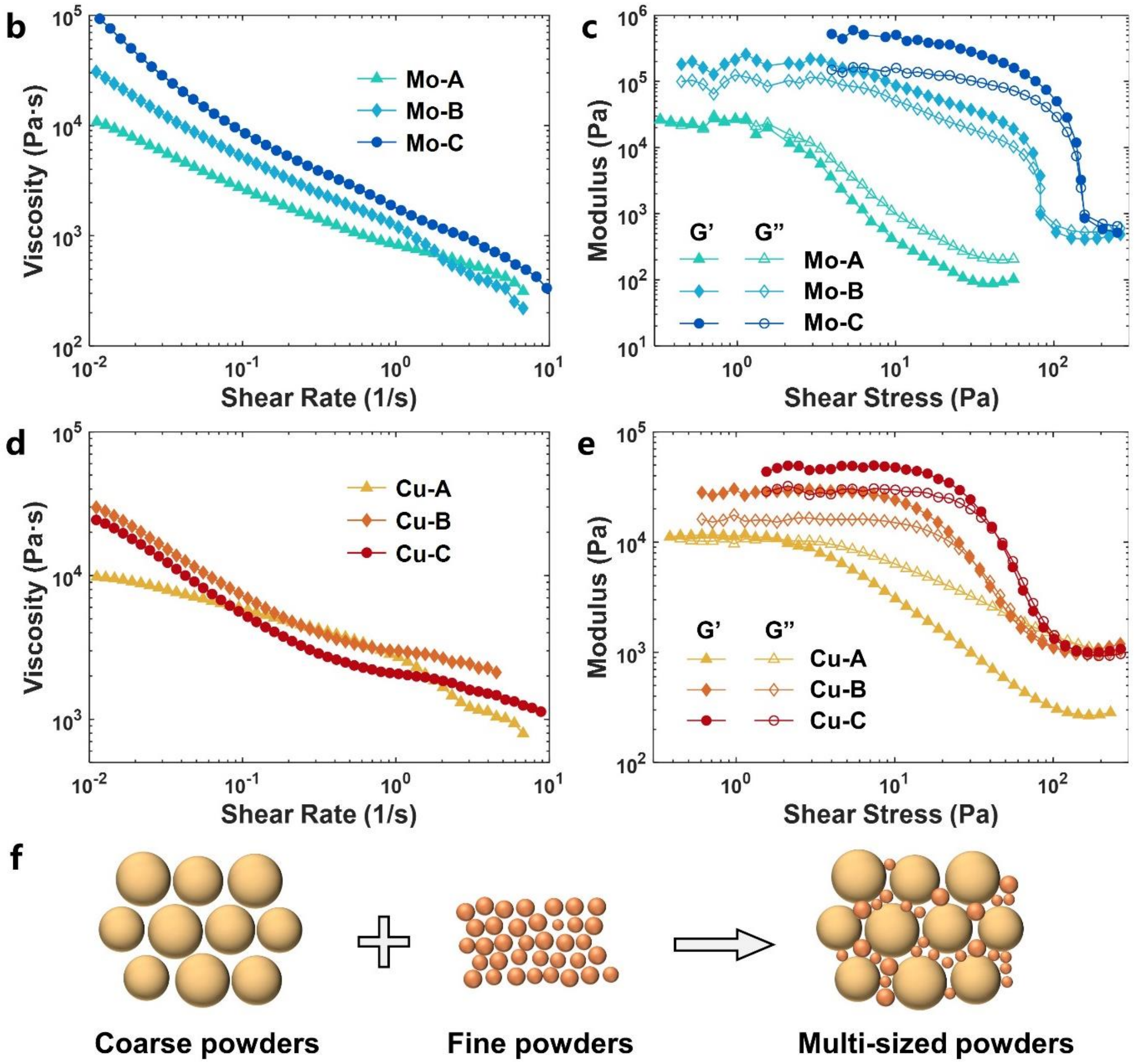

Figure S4. Rheological behavior of metal-tar inks. a) Table of ink compositions. In the first column, the Mo-tar inks differ in the weight percentage of metal powders. In the second column, the Cu-tar inks differ in the particle sizes. b,c) Comparison of rheological behavior of Mo-tar inks. d,e) Comparison of rheological behavior of Cu-tar inks. f) Schematic showing the packing of multi-sized powders.

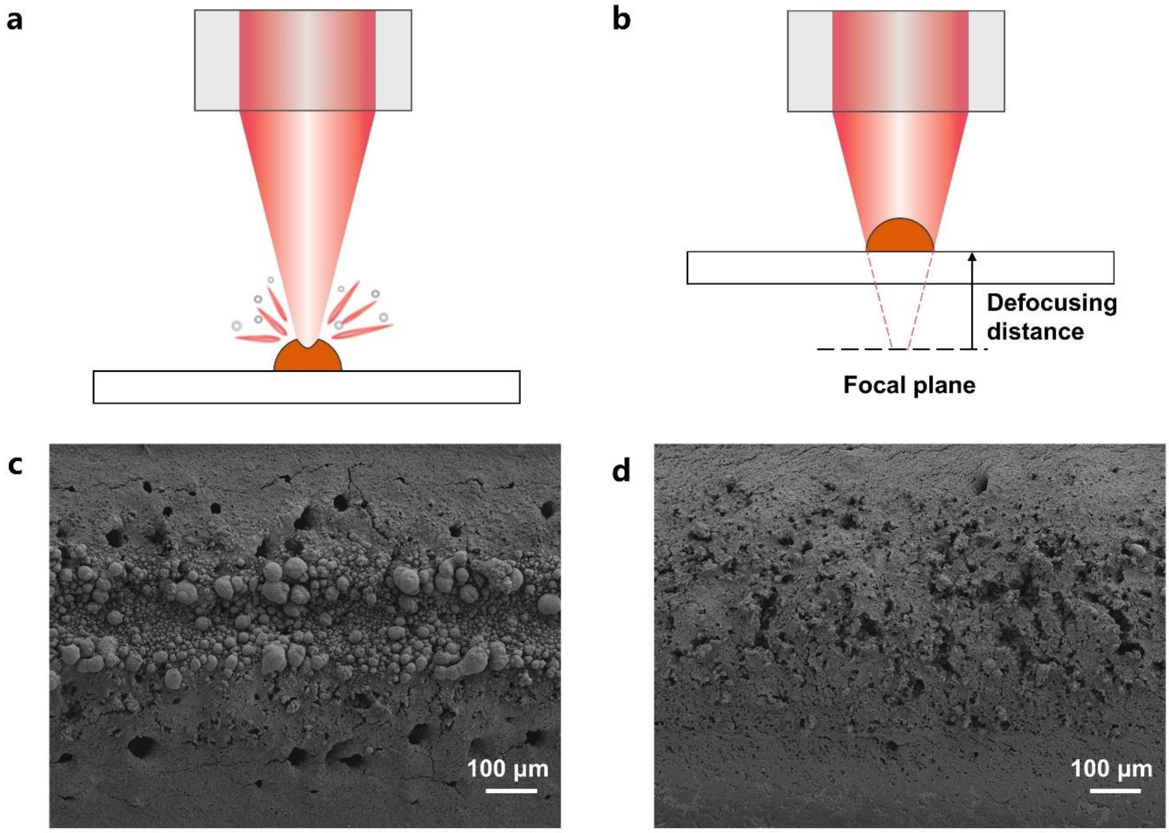

Figure S5. Schematics showing the processing conditions of a) laser focusing and b) defocusing. SEM images showing the surface morphology of printed Cu-tar filament with c) focused scanning and d) defocused scanning. Laser parameters: 75 mm/min - 7 W.

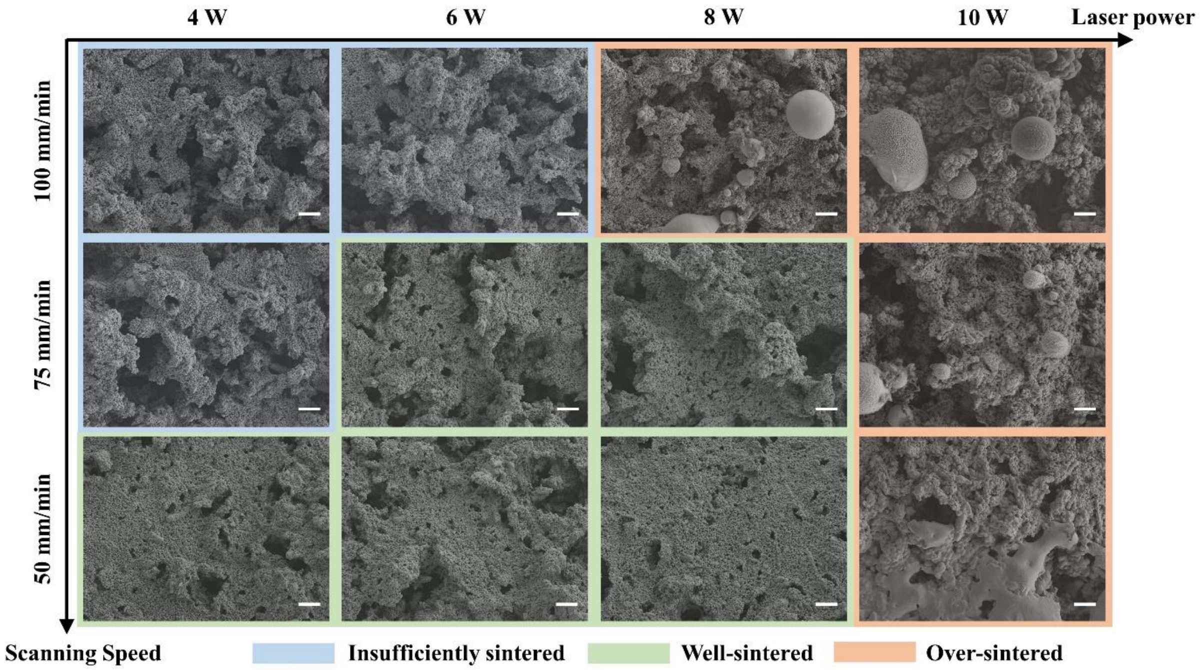

Figure S6. SEM images of sintered Cu-tar filaments at different laser powers and scanning speeds. Scale bar: 10 μm.

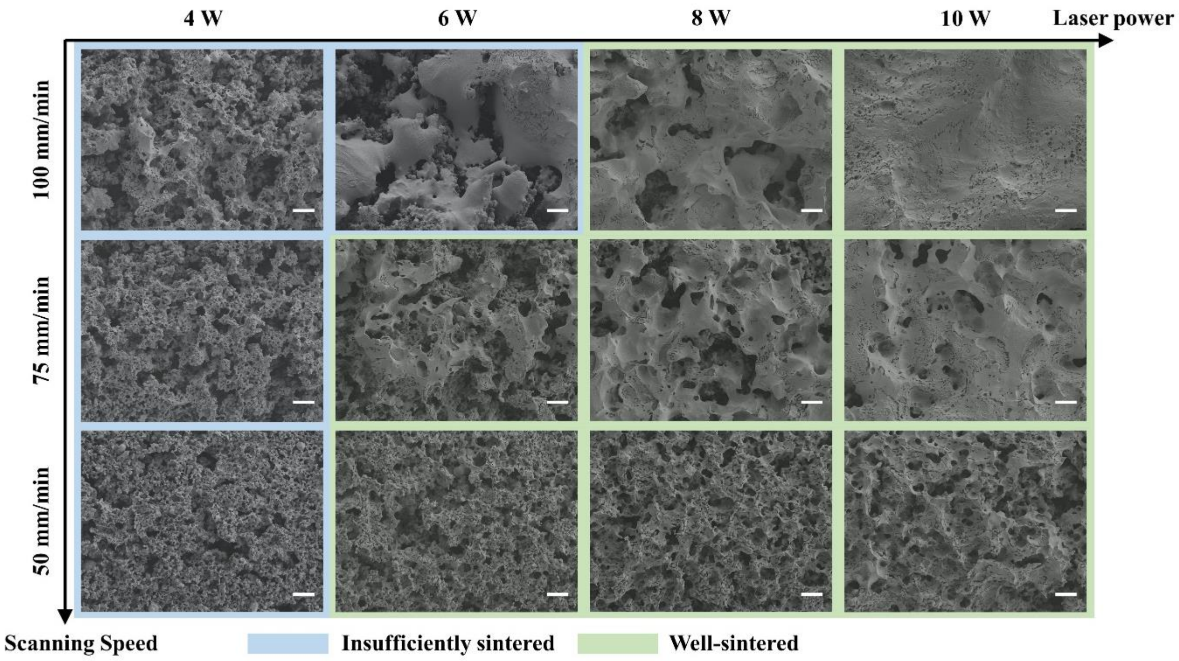

Figure S7. SEM images of sintered Mo-tar filaments at different laser powers and scanning speeds. Scale bar: 20 μm.

Design and Measurement of Hemispherical Antenna

The hemispherical antenna is designed as three spatial spirals with an angle difference of 120˚. The spiral equation is expressed as

$$\begin{cases} x(u) = R \cdot \cos\left(\frac{u}{4N}\right)\cos(u) \\ y(u) = -R \cdot \cos\left(\frac{u}{4N}\right)\sin(u) \qquad u \in (0, 2\pi N) \\ z(u) = R \cdot \sin\left(\frac{u}{4N}\right) \end{cases} \qquad (S1)$$

where *N* denotes the number of spiral turns. The spirals start 2 mm above the ground plane, and are connected to the top ring with 4 mm diameter (**Figure S5**). The antenna is fed by a coaxial transmission line which is connected to one of the spirals at the bottom.

The Wheeler cap method is adopted to measure the radiation efficiency.[4] The free-space input impedance of the antenna R_{fs} is firstly measured. Then a hemispherical metallic cap with radius $r = \lambda / 2\pi$ (the Wheeler cap) is placed over the antenna and secured to the ground, in which case the input impedance is measured to be R_{cap}. The input impedance of the antenna is well approximated by a parallel RLC circuit. Therefore, the radiation efficiency is calculated as

$$\eta = 1 - \frac{R_{fs}}{R_{cap}} \qquad (S2)$$

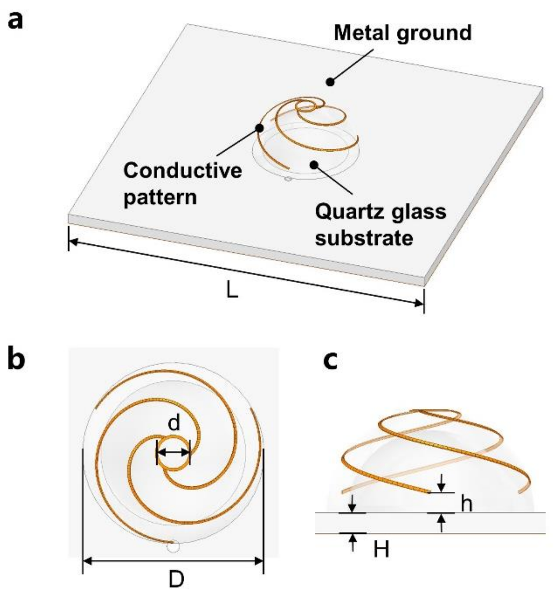

d

Design Parameter	Value
L	70 mm
H	2 mm
D	20 mm
d	4 mm
h	2 mm
Hemisphere thickness	2 mm
Conductive line width	0.4 mm
Conductivity of the pattern	3×10^5 S/m
Dielectric constant of substrate	3.6
N (Number of turns)	0.9

Figure S8. a) 3D view, b) top view and c) front view of the hemispherical antenna. d) Detailed design parameters.

Table S2. Performance comparison of electrically small hemispherical antennas fabricated by different methods.

**Fabrication method**	**Total processing time**	**Antenna design**	**ka**	**Center Frequency (GHz)**	**Half Power Bandwidth**	**Efficiency**	**Quality Factor**
Pad-printing[5]	> 15 mins	3-arm spiral	0.32	1.28	7.1%	57%	28.2
		3-arm spiral	0.43	1.71	23.1%	55%	8.7
Direct transfer printing[6]	hours	3-arm spiral	0.31	1.52	5.3%	69%	45.7
		3-arm spiral	0.56	2.70	14.2%	88%	14.5
Silver ink printing[7]	> 3 hours	8-arm meander line	0.46	1.73	15.2%	71%	13.1
		4-arm meander line	0.21	0.79	6.3%	14%	31.9
DIW-TMLS (this work)	< 5 mins	3-arm spiral	0.48	2.29	10.5%	56%	19.0

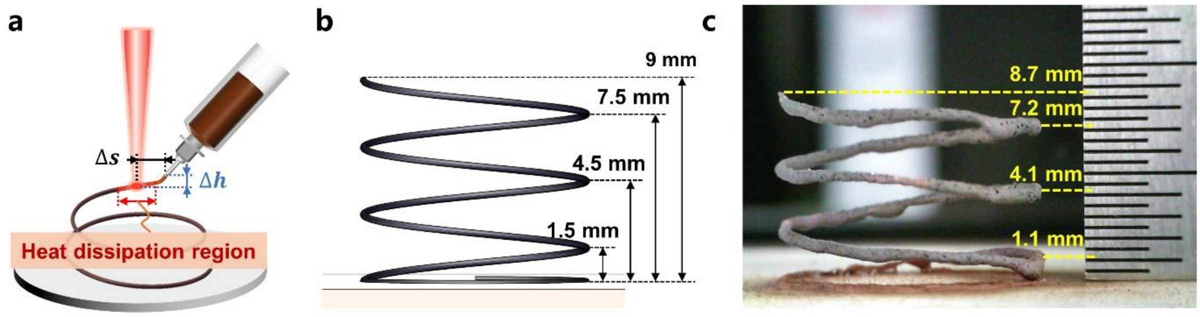

Figure S9. a) Schematic showing the effect of laser-nozzle gap on the forming accuracy during mid-air sintering. b) Helical trajectory of the printing nozzle. c) Photograph showing the dimensions of the printed helical structure.

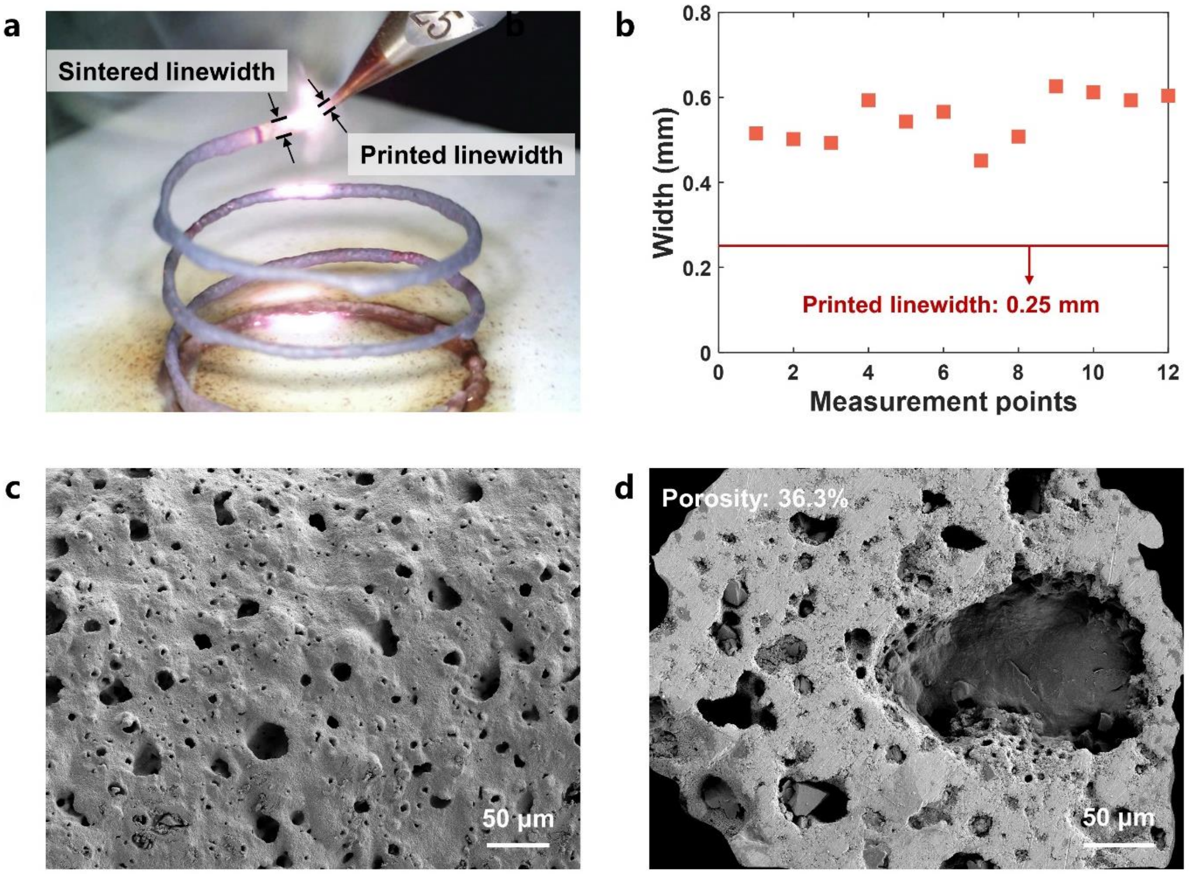

Figure S10. a) Photograph of the mid-air sintering process showing the volumn increase after sintering. b) Measurement results of the sintered linewidth. 12 measurement points were evenly selected along the helix structure. c) SEM image showing the porous surface of the sintered structure. d) SEM image showing the cross-section of the sintered structure.

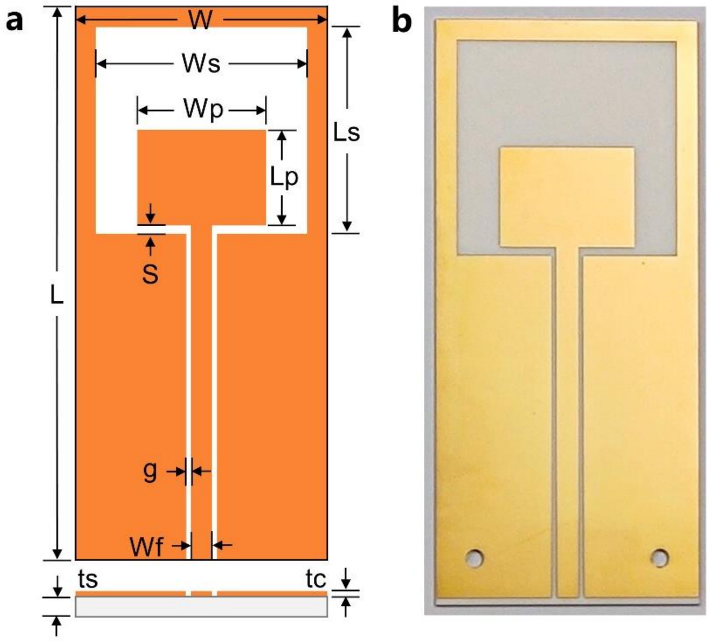

c

Design Parameter	Value (mm)
L	117.60
W	53.60
Ls	44.00
Ws	45.00
Lp	20.32
Wp	27.40
S	1.80
Wf	4.50
g	1.00
ts	1.00
tc	0.035

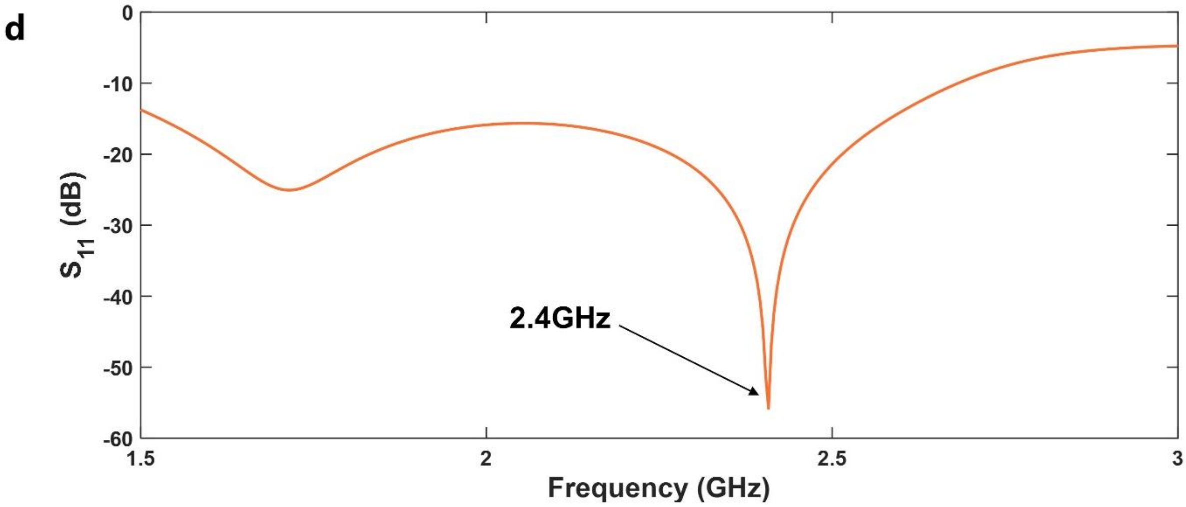

Figure S11. a) Schematic and b) photograph of the interrogation antenna. c) Detailed design parameters. d) Simulated reflection coefficient of the interrogation antenna.

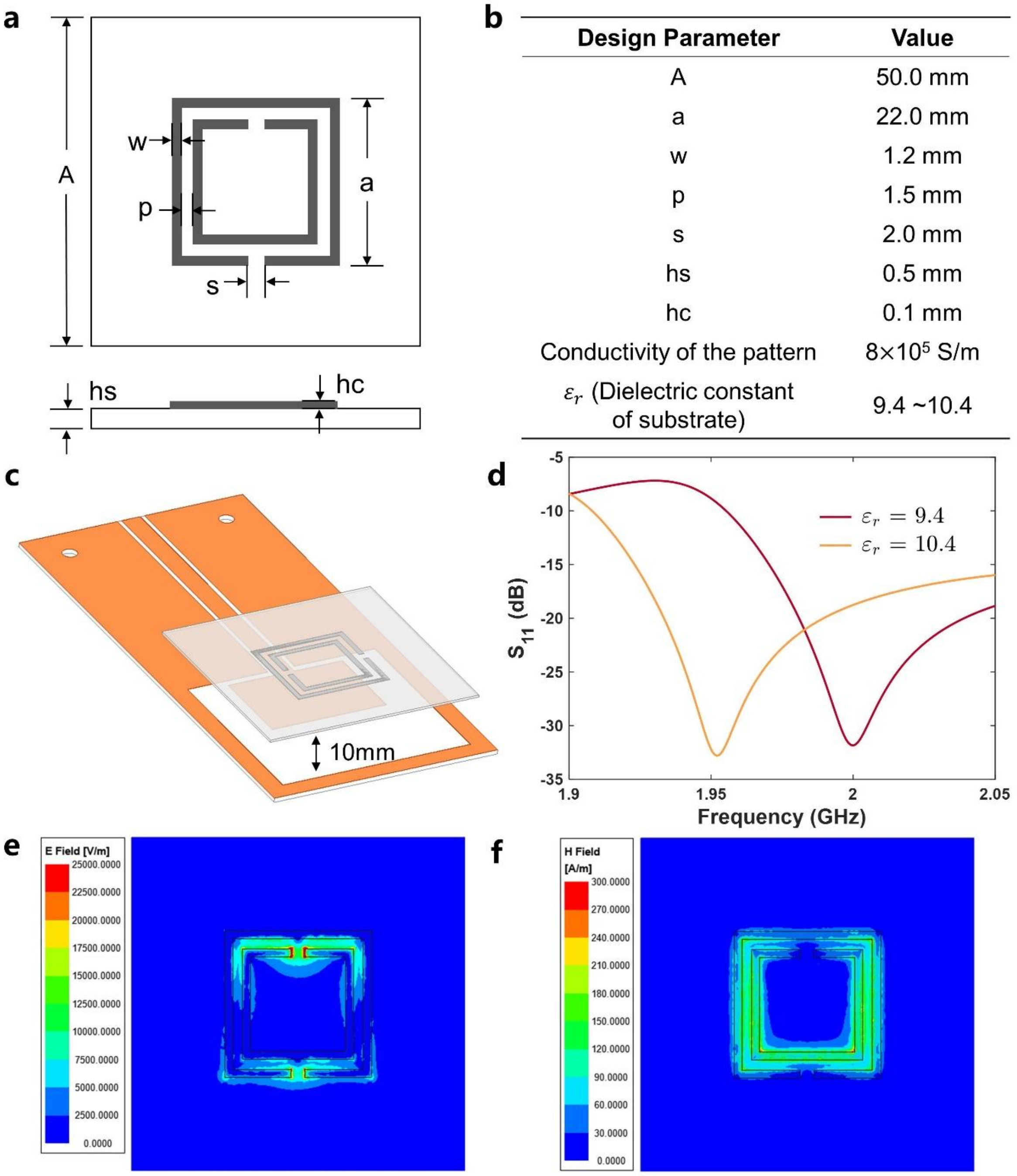

Figure S12. a) Schematic and b) design parameters of the SRR sensor. c) Schematic of wireless interrogation in simulation. d) Simulated reflection coefficient of the SRR sensor. e) Simulated electric field distribution and f) magnetic field distribution in the sensor.

Supporting Videos

Video S1. Fabrication of hemispherical spiral antenna.

Video S2. Mid-air sintering of freestanding helical structure.

References:

[1] R. D. Farahani, L. L. Lebel, D. Therriault, *J. Micromechanics Microengineering* 2014, *24*, 055020.

[2] X. Fan, Y. Fei, L. Chen, W. Li, *Energy and Fuels* **2017**, *31*, 4694.

[3] W. Zhang, J. T. Andersson, H. J. Räder, K. Müllen, *Carbon N. Y.* **2015**, *95*, 672.

[4] W. E. McKinzie, *IEEE Antennas Propag. Soc. AP-S Int. Symp.* **1997**, *1*, 542.

[5] H. Wu, S. W. Chiang, C. Yang, Z. Lin, J. Liu, K. S. Moon, F. Kang, B. Li, C. P. Wong, B. Xu, *PLoS One* **2015**, *10*, 1.

[6] C. Pfeiffer, X. Xu, S. R. Forrest, A. Grbic, *Adv. Mater.* **2012**, *24*, 1166.

[7] J. J. Adams, E. B. Duoss, T. F. Malkowski, M. J. Motala, B. Y. Ahn, R. G. Nuzzo, J. T. Bernhard, J. A. Lewis, *Adv. Mater.* **2011**, *23*, 1335.
